# Evaluating sediment and water sampling methods for the estimation of deep-sea biodiversity using environmental DNA

**DOI:** 10.1038/s41598-021-86396-8

**Published:** 2021-04-12

**Authors:** Miriam I. Brandt, Florence Pradillon, Blandine Trouche, Nicolas Henry, Cathy Liautard-Haag, Marie-Anne Cambon-Bonavita, Valérie Cueff-Gauchard, Patrick Wincker, Caroline Belser, Julie Poulain, Sophie Arnaud-Haond, Daniela Zeppilli

**Affiliations:** 1 MARBEC, IFREMER, IRD, CNRS, Univ Montpellier, Sète, France; 2 Centre Brest, Laboratoire Environnement Profond (REM/EEP/LEP), IFREMER, CS10070, 29280 Plouzané, France; 3 IFREMER, CNRS, Laboratoire de Microbiologie Des Environnements Extrêmes (LM2E), Univ Brest, Plouzané, France; 4 CNRS, Station Biologique de Roscoff, AD2M, UMR 7144, Sorbonne University, 29680 Roscoff, France; 5 Génomique Métabolique, Genoscope, Institut François Jacob, CEA, CNRS, Univ of Évry, Paris-Saclay University, 91057 Evry, France

**Keywords:** Biodiversity, Ecological genetics, Microbial ecology, Molecular ecology

## Abstract

Despite representing one of the largest biomes on earth, biodiversity of the deep seafloor is still poorly known. Environmental DNA metabarcoding offers prospects for fast inventories and surveys, yet requires standardized sampling approaches and careful choice of environmental substrate. Here, we aimed to optimize the genetic assessment of prokaryote (16S), protistan (18S V4), and metazoan (18S V1–V2, COI) communities, by evaluating sampling strategies for sediment and aboveground water, deployed simultaneously at one deep-sea site. For sediment, while size-class sorting through sieving had no significant effect on total detected alpha diversity and resolved similar taxonomic compositions at the phylum level for all markers studied, it effectively increased the detection of meiofauna phyla. For water, large volumes obtained from an in situ pump (~ 6000 L) detected significantly more metazoan diversity than 7.5 L collected in sampling boxes. However, the pump being limited by larger mesh sizes (> 20 µm), only captured a fraction of microbial diversity, while sampling boxes allowed access to the pico- and nanoplankton. More importantly, communities characterized by aboveground water samples significantly differed from those characterized by sediment, whatever volume used, and both sample types only shared between 3 and 8% of molecular units. Together, these results underline that sediment sieving may be recommended when targeting metazoans, and aboveground water does not represent an alternative to sediment sampling for inventories of benthic diversity.

## Introduction

Environmental DNA (eDNA) metabarcoding is an increasingly used tool for non-invasive and rapid biodiversity surveys and impact assessments. Using high-throughput sequencing (HTS) and bioinformatic processing, target organisms are detected using their DNA directly extracted from soil, water, or air samples^1^. Covering more than 50% of Planet Earth, the deep seafloor is mostly comprised of sedimentary habitats, characterised by a predominance of small organisms^2,3^ difficult to identify based on morphological features^4^, and by high local and regional diversity^5–7^. Given its increased time-efficiency and its wide taxonomic applicability, eDNA metabarcoding is thus a good candidate for large-scale biodiversity surveys and Environmental Impact Assessments in the deep-sea biome.

Size-class sorting such as sieving or elutriation is usually performed on sediment samples in order to split the organisms by size and facilitate morphological characterization of meiofauna and macrofauna. For metabarcoding approaches, it also has the advantage of limiting the over dominance of large organisms, which may produce higher amounts of DNA template, resulting in an incomplete detection of small and abundant taxa. However, sieving requires large volumes of sediment, is very time-consuming, and previous studies have found that the use of non-sieved material does not significantly alter metazoan diversity patterns^8^, suggesting that dominance of large (and often rare) taxa in the DNA extract does not result in important biases. Besides, for logistic reasons, the use of non-sieved sediment samples is preferable to (1) minimize on-board processing time, (2) minimize risks of contamination, and (3) facilitate other future applications (e.g., avoid RNA degradation, avoid losing the extracellular DNA compartment).

Finally, studies from various marine habitats have reported that benthic taxa could be found in aboveground water (overlying water layer to 6.5 m above seafloor), possibly due to sediment resuspension and transport, but also to active dispersal^9–11^. Application of eDNA metabarcoding on deep-sea aboveground water could thus be a convenient alternative to surface sediment collection, as it involves simplified sample processing and shipping, while additionally allowing investigating benthopelagic diversity and dispersal capacities of benthic organisms. However, distance above seafloor has been variable (0.5–6.5 m) among studies^9–11^, and so has the water volume sampled (12–1000 L). As the latter is a crucial aspect for efficient species detection^12^, it remains unclear whether small volumes (< 10 L) are sufficient to obtain comprehensive species inventories in the deep-sea.

To evaluate the effect of sampling strategy on eDNA metabarcoding inventories targeting prokaryotes (16S V4-V5), unicellular eukaryotes (18S V4), and metazoans (18S V1–V2, COI) from deep-sea sediment and aboveground water, we compared biodiversity inventories resulting from (1) sieved versus unsieved sediment and (2) on-board filtration of 7.5 L of water collected with a sterile sampling box versus in situ filtration of large volumes (~ 6000 L) using a newly-developed pump.

## Results

### High-throughput sequencing results

A total of 26 million COI reads, 19 million raw 18S V1-V2 reads,, 14 million 18S V4 reads, and 17 million 16S V4–V5 reads were obtained from three Illumina HiSeq runs of amplicon libraries built from pooled triplicate PCRs of 22 environmental samples, 2 extraction blanks, and 4–6 PCR blanks (Supplementary Table S4 online). The in situ pump yielded less raw reads for COI and 16S (Supplementary Fig. S1 online, F = 4.02–14.4, *p* = 0.0003–0.03), while more raw reads were recovered from both water sampling methods with 18S V4 (F = 6.5, *p* = 0.007). Water samples generally yielded fewer raw clusters (F = 5.1–35.1, *p* = 3.2 × 10^−6^–0.02), except for 18S V4 where numbers were comparable across sample types (Supplementary Fig. S1 online).

Bioinformatic processing (quality filtering, error correction, chimera removal, and clustering for metazoans) reduced read numbers to 20 million for COI, 12 million for 18S V1–V2, 11 million for 18S V4, and 10 million for 16S V4–V5, resulting in 10,351 and 17,608 raw OTUs for COI and 18S V1–V2 respectively; 35,538 raw 18S V4 ASVs, and 62,646 raw 16S ASVs (Supplementary Table S4 online). For eukaryote markers, 17–55% of the raw reads remained in PCR blanks after bioinformatic processing, while 50–75% remained in extraction blanks and 52–87% in true samples. In contrast, with 16S, these values were at 87% for PCR blanks, 67% for extraction blanks, and 29–73% for true samples. Thus, negative control samples accounted for 7–13% of bioinformatically processed reads with eukaryotes, compared to 27% with prokaryotes. The vast majority of 16S reads generated by negative controls belonged to a common contaminant of *Phusion* polymerase kits, which is well amplified in low concentration samples such as negative controls. These reads however accounted for < 1% of 16S ASVs. After data refining (decontamination, removal of all control samples and of all unassigned or non-target clusters), rarefaction curves showed a plateau was reached for all samples except in situ pump samples with microbial loci and sediment samples with 18S V4, suggesting that not all protist and prokaryote diversity was captured at this sequencing depth in these samples (Supplementary Fig. S1 online). Refined datasets contained 7 million reads for prokaryotes and between 4.8 and 8.5 million target reads for eukaryotes, delivering 38,816 prokaryote ASVs (16S V4–V5), 8031 protist ASVs (18S V4), and 2,319 (COI) and 1,460 (18S V1–V2) LULU curated metazoan OTUs (Table S4). For COI, while only 1.2% of metazoan OTUs were unassigned at phylum-level, 57% had BLAST hit identities < 80%, i.e. unreliable at phylum-level. For 18S, 7% (18S V4) to 16% (18S V1-V2) of final ASVs/OTUs were unassigned at phylum-level, but only 12% (18S V4) and 13% (18S V1-V2) had BLAST hit identities < 86%. For 16S, 0.9% and 3% of ASVs had no or unreliable phylum-level assignments, respectively.

### Alpha diversity between sampling methods

**Figure 1:**
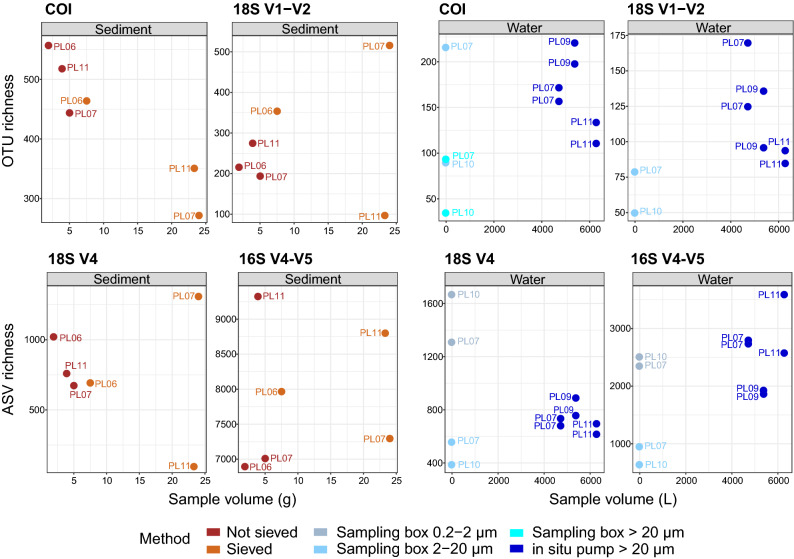
Numbers of metazoan OTUs (COI, 18S V1–V2), unicellular eukaryote (18S V4) and prokaryote (16S V4–V5) ASVs recovered by deep-sea sediment (brown) and aboveground water (blue), using for each sample type two sampling methods based on varying amounts of starting material. Sediment was either sieved through 5 mesh sizes to size-sort organisms prior DNA extraction, or DNA was extracted directly from crude sediment samples. Water was collected with a 7.5 L sampling box, allowing recovery of up to two size classes per taxonomic compartment, or sampled in large volumes with an in situ pump. Cluster abundances were calculated on rarefied datasets.

The number of recovered molecular clusters significantly differed with sampling method, water samples detecting less diversity than sediment samples for metazoans and prokaryotes (COI: F = 20.1, *p* = 4.4 × 10^−5^, 18S V1–V2: F = 6.5, *p* = 0.01, 16S: F = 56.0, *p* = 3 × 10^−7^), but both sample types recovering similar levels of protist diversity (18S V4: F = 2.9, *p* = 0.07, Fig. 1). Sieved and unsieved sediment resulted in statistically comparable total cluster numbers in all loci investigated (Table 1) although, for metazoans detected with 18S V1–V2, this lack of significance was likely due to the very low yield observed in one sieved sample (PL11), as the other sieved samples detected considerably more OTUs (Fig. 1). For metazoans detected with COI, sieved samples tended to detect less OTUs although being based on larger sediment volumes (pool of 5 DNA extracts). The number of total recovered OTUs did not differ significantly between the water sampling box and the in situ pump for metazoans (COI, 18S V1–V2), but differences were observed for unicellular eukaryotes (18S V4) and prokaryotes (16S V4–V5) depending on the sampling box size fraction (Table 1), with the smallest fraction (0.2–2 µm) detecting more ASVs (Fig. 1).

**Table 1:** Effect of sampling method on cluster richness and community structure for the 4 studied genes.

Pairwise comparison	Cluster richness				Community differentiation			
	COI	18S V1–V2	18S V4	16S V4–V5	COI	18S V1–V2	18S V4	16S V4–V5
Not sieved/Sieved*	0.26	0.64	0.99	1.0	0.4, R^2^ = 0.22	0.1, R^2^ = 0.22	0.1, R^2^ = 0.26	0.1, R^2^ = 0.42
Sampling box 0.2–2 µm/not sieved*	na	na	0.26	** < 0.0001**	na	na	0.1, R^2^ = 0.64	0.1, R^2^ = 0.87
Sampling box 2–20 µm/Not sieved*	** < 0.0001**	0.10	0.63	** < 0.0001**	0.1, R^2^ = 0.51	0.1, R^2^ = 0.46	0.1, R^2^ = 0.57	0.1, R^2^ = 0.89
Sampling box > 20 µm/not sieved*	** < 0.0001**	na	na	na	0.1, R^2^ = 0.50	na	na	na
Sampling box 0.2–2 µm/sieved*	na	na	0.10	** < 0.0001**	na	na	0.1, R^2^ = 0.56	0.1, R^2^ = 0.88
Sampling box 2–20 µm/sieved*	**0.01**	**0.02**	0.86	** < 0.0001**	0.1, R^2^ = 0.44	0.1, R^2^ = 0.43	0.1, R^2^ = 0.49	0.1, R^2^ = 0.89
Sampling box > 20 µm/sieved*	**0.0001**	na	na	na	0.1, R^2^ = 0.41	na	na	
In situ pump/not sieved	** < 0.0001**	0.13	0.99	** < 0.0001**	**0.01**, R^2^ = 0.32	**0.01**, R^2^ = 0.29	**0.02**, R^2^ = 0.45	**0.01**, R^2^ = 0.78
In situ pump/sieved	**0.0001**	**0.002**	1.00	** < 0.0001**	**0.01**, R^2^ = 0.28	**0.01**, R^2^ = 0.28	**0.01**, R^2^ = 0.38	**0.007**, R^2^ = 0.78
Sampling box 0.2–2 µm/in situ pump	na	na	**0.04**	1.0	na	na	**0.03**, R^2^ = 0.49	**0.04**, R^2^ = 0.74
Sampling box 2–20 µm/in situ pump	0.99	0.70	0.79	**0.001**	**0.03**, R^2^ = 0.25	**0.03**, R^2^ = 0.25	**0.04**, R^2^ = 0.43	**0.04**, R^2^ = 0.59
Sampling box > 20 µm/in situ pump	0.10	na	na	na	**0.04**, R = 0.21	na	na	na
Sampling box 0.2–2 µm/Sampling box 2–20 µm*	na	na	**0.03**	**0.007**	na	na	0.3, R^2^ = 0.47	0.3, R^2^ = 0.89
Sampling box 0.2–2 µm/Sampling box > 20 µm*	na	na	na	na	na	na	na	na
Sampling box 2–20 µm/Sampling box > 20 µm*	0.26	na	na	na	0.3, R^2^ = 0.45	na	na	na

Pairwise comparisons of ANOVAs of ASV/OTU richness performed using rarefied datasets. Pairwise PERMANOVAs were performed on rarefied datasets using Jaccard distances for metazoans and Bray–Curtis distances for 18S V4 and 16S V4–V5. Significance was evaluated by permuting 999 times when possible, and comparisons where this was not possible are marked by *. Significant *p* values are in bold. R^2^: R-squared.

Differences in total recovered diversity were not solely a result of differences in sample volume. Indeed, sieved samples, based on 3–6 times more sediment, did not consistently detect more diversity (Fig. 1). Similarly, the in situ pump, although sampling ~ 800 times more water than the sampling box, did not consistently detect more diversity than any size fraction of the sampling box (Fig. 1).

**Figure 2:**
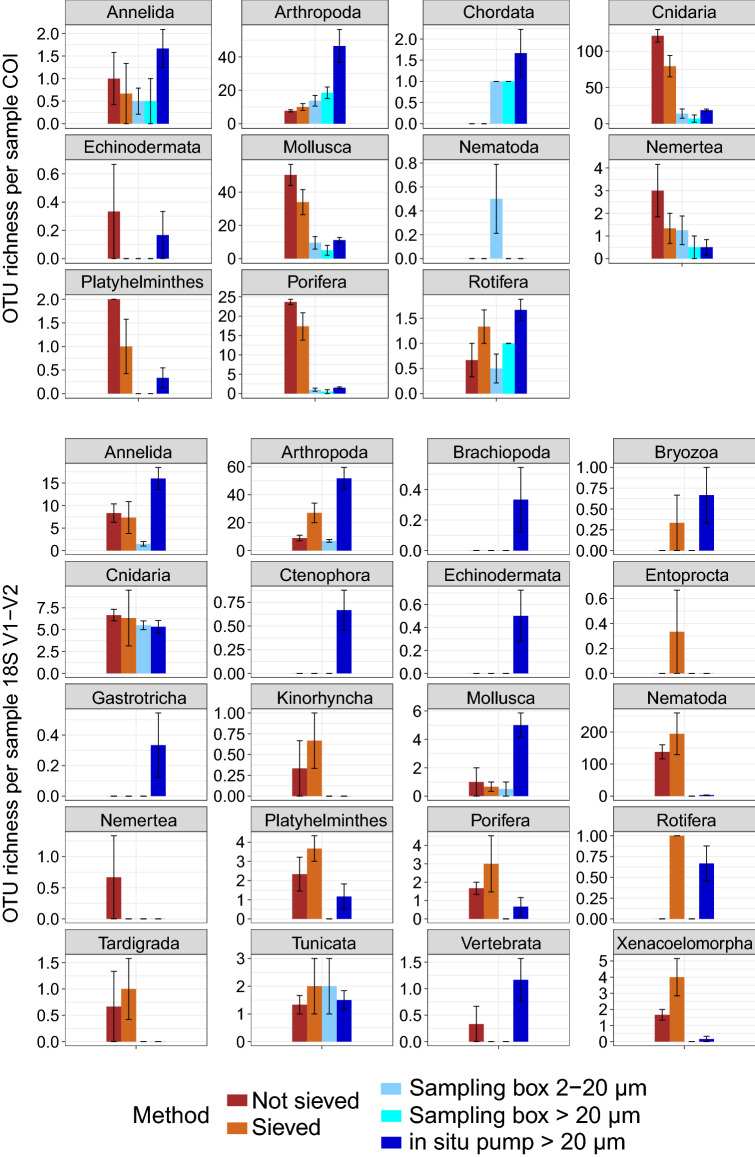
Mean numbers (± SE) of metazoan COI and 18S V1-V2 OTUs detected in target phyla for sediment (brown) and water (blue), using two sampling methods for both sample types. Sediment was either sieved to size-sort organisms prior DNA extraction, or DNA was extracted directly from crude sediment samples. Water was collected with a 7.5 L sampling box, allowing recovery of two size classes, or sampled in large volumes with an in situ pump. OTU numbers were calculated on rarefied datasets.

Recovered diversity among sample types strongly differed depending on phylum (Fig. 2). For metazoans, water samples led to the detection of significantly higher numbers of OTUs than sediment samples for Arthropoda, Chordata (COI, t-tests, *p* = 0.006–0.01) and Mollusca (18S V1–V2, t-test, *p* = 0.03), and some phyla like Brachiopoda, Ctenophora, Echinodermata, or Gastrotricha, were only detected in water samples (Fig. 2). In contrast, phyla such as Platyhelminthes, Porifera (COI, 18S V1–V2, t-tests, *p* = 0.001–0.04), Kinorhyncha, Nematoda, Tardigrada, or Xenacoelomorpha (18S V1–V2, t-tests, *p* = 0.001–0.04) produced significantly more OTUs in sediment than water samples (Fig. 2). Similarly, some protistan groups, such as the Acantharea, Chlorophyta, Dinophyceae, or Syndiniales (t-tests, *p* = 0.002–0.03) were predominant in water samples (Supplementary Fig. S2 online), while others were significantly more diverse in sediment, e.g., Apicomplexa, Filosa groups, Ciliophora, Labyrinthulea, RAD-B (t-tests, *p* = 0.001–0.04). For prokaryotes, most lineages were predominant in sediment (t-tests, *p* = 2.2 × 10^−7^–0.02, e.g., Archaea, Acidobacteria, Actinobacteria, Bacteroidetes, Chloroflexi, Delta-, Gamma-proteobacteria, Gemmatimonadetes, Latescibacteria, Hydrogenedentes, Nitrospirae, Planctomycetes), and only Cyanobacteria were significantly more diverse in water samples (t-test, *p* = 0.001).

For sediment, recovered levels of alpha diversity among sampling methods also varied by phyla and organism size class (Fig. 2). For meiofauna phyla, best detected with 18S V1–V2, more OTUs were detected from sieved than from unsieved sediment (Kinorhyncha, Nematoda, Platyhelminthes, Rotifera, Tardigrada, Xenacoelomorpha), although this difference was not significant, likely due to the low sample size. However, differences in alpha diversity among sampling methods were not only a result of differences in sample volume, as some unsieved samples yielded similar or greater numbers of OTUs than sieved samples for many phyla (Supplementary Fig. S3 online). Sieved and unsieved sediment detected comparable ASV numbers in most microbial groups, except the Chrysophyceae, Actinobacteria, Cyanobacteria, Gammaproteobacteria, Nanoarchaeaeota (Supplementary Fig. S2 online, paired t-tests, *p* = 0.02–0.04).

Water sampling methods strongly differed in terms of recovered alpha diversity depending on taxonomic compartment. The in situ pump generally detected more metazoan diversity than the sampling box, and phyla such as Brachiopoda, Ctenophora, and Echinodermata were only detected by the pump (Fig. 2). However, the in situ pump detected significantly more ASVs than the sampling box only in some taxonomic groups for protists (Bacillariophyta, Oomycota, Phaeodarea) and prokaryotes (e.g., Bacteroidetes, Chlamydiae, Firmicutes, Tenericutes, Lentisphaerae, and Delta-, Gammaproteobacteria, see Supplementary Fig. S2 online, t-tests, *p* = 9 × 10^−5^–0.003). Other clades were significantly more diverse in the sampling box (e.g., the protist groups Haptophyta, Picozoa, Telonemia, and the Cyanobacteria, t-tests, *p* = 0.002–0.02). With the sampling box, the smallest size fraction (0.2–2 µm) allowed recovering more alpha diversity in all microbial groups than the larger size fraction (2–20 µm). This difference was significant only for Chlorophyta, Labyrinthulea, Chloroflexi, and Verrucomicrobia (paired t-tests, *p* = 0.01–0.03), although non-significant comparisons may result from the limited number of samples available. The two size fractions available with the sampling box for COI (2–20 µm, > 20 µm) did not reveal differences in diversity recovery with size class, as most phyla were detected equally well in both (Fig. 2).

### Effect of sampling method on community structures

Community compositions significantly differed among sampling methods for all investigated loci (COI: pseudo-F = 2.3, *p* = 0.001; 18S V1–V2: pseudo-F = 2.3, *p* = 0.001, 18S V4: pseudo-F = 4.1, *p* = 0.001, 16S: pseudo-F = 18.3, *p* = 0.001) and sampling method accounted for 41–45% of variation among samples for metazoans (COI, 18S V1–V2), 60% for protists (18S V4), and 87% for prokaryotes (16S).

**Figure 3:**
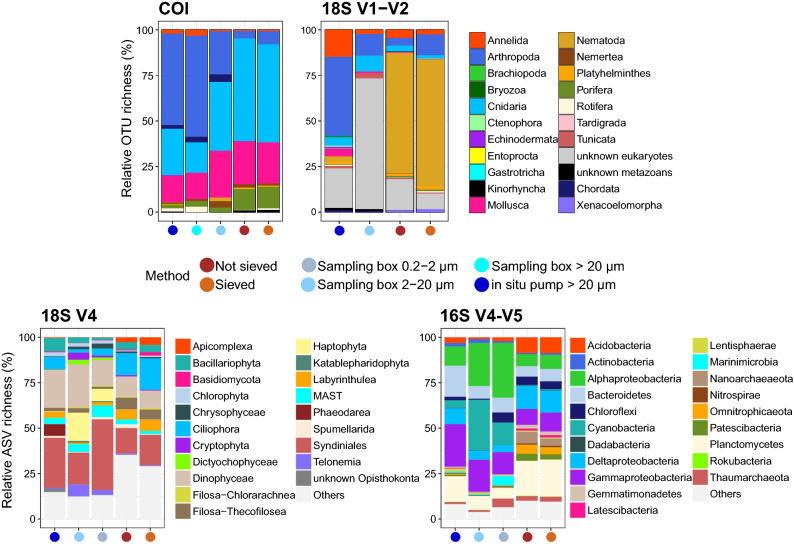
Patterns of relative cluster abundance resolved by eDNA metabarcoding of deep-sea sediment (brown) and aboveground water (blue), using two sampling methods for both sample types, and using four barcode markers targeting metazoans (COI, 18S V1–V2), micro-eukaryotes (18S V4), and prokaryotes (16S V4–V5). Sediment was either sieved to size-sort organisms prior DNA extraction, or DNA was extracted directly from crude sediment samples. Water was collected with a 7.5 L sampling box, allowing recovery of up to two size classes per taxonomic compartment, or sampled in large volumes with an in situ pump. Top 20 most abundant taxa are displayed for microbial groups.

Pairwise PERMANOVAs showed that community structures differed most strongly among sample types (water or sediment, R^2^ = 0.28–0.89), although not all pairwise comparisons were significant, likely due to the limited number of samples available for the sampling box (Table 1). Relative taxonomic compositions revealed by aboveground water samples differed from sediment samples, with higher proportions of arthropods, chordates, annelids, and tunicates in the water samples, while nematodes, poriferans, platyhelminths, and xenacoelomorphs were predominant in sediment samples (Fig. 3 COI and 18S V1–V2). Similarly, protist diversity in aboveground water samples was dominated by Dinophyceae, Haptophyta, Phaeodarea, Syndiniales, and to a lesser extent Bacillariophyta and Telonemia, while apicomplexans, ciliates, filosans, and labyrinthuleans represented higher proportions of diversity in sediment samples (Fig. 3 18S V4). For prokaryotes, aboveground water communities were characterised by Alphaproteobacteria, Cyanobacteria, and Gammaproteobacteria, while Acidobacteria, Deltaproteobacteria, Archeae, Latescibacteria, and Planctomycetes showed higher diversity in sediment (Fig. 3 16S V4–V5).

**Figure 4:**
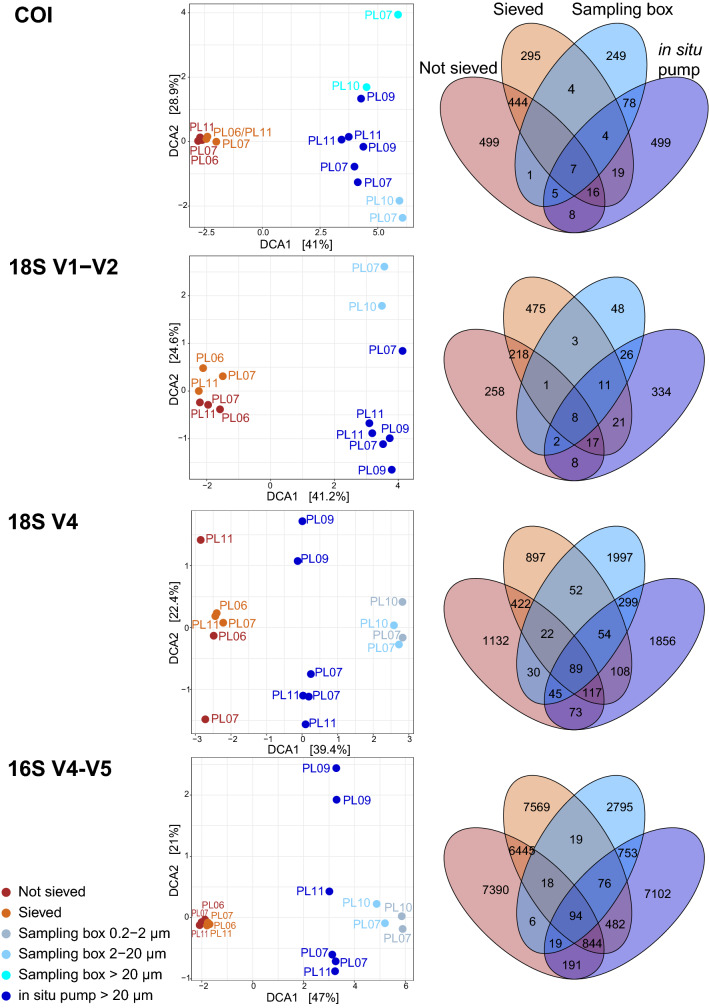
Detrended Correspondence Analyses (DCA) ordinations (left) and Venn diagrams (right), showing differences in community compositions detected by deep-sea sediment (brown) and aboveground water (blue) for metazoans (COI and 18S V1–V2), micro-eukaryotes (18S V4), and prokaryotes (16S V4–V5). Community segregation is strongest between sample types, but also among target size class in the water samples. Sediment was either sieved to size-sort organisms prior DNA extraction, or DNA was extracted directly from crude sediment samples. Water was collected with a 7.5 L sampling box, allowing recovery of two size classes in each taxonomic compartment, or sampled in large volumes with an in situ pump.

Only 3% (COI), 5% (18S V1–V2), 8% (18S V4), and 5% (16S) of clusters were shared between sediment and water samples, resulting in strong segregation in ordinations (Fig. 4). For metazoans, taxa shared among water and sediment samples were mostly assigned to hydrozoans (COI, 28%, 18S, 7%), copepods (COI, 6%, 18S, 20%), gastropods (COI, 31%), demosponges (COI, 6%), or polychaetes (18S, 10%), and chromadorean nematodes (18S, 11%). For protists, ASVs shared among sample types primarily belonged to the Syndiniales (39%), but other taxa included dinophyceans (11%), filosans (9%), labyrinthuleans (5%), and bacillariophytes (6%). For prokaryotes, ASVs shared across sample types were predominantly belonging to the Proteobacteria (Gamma, 19%, Alpha, 10%, Delta, 8%), Bacteroidetes (15%), or Planctomycetes (16%).

Sediment processing did not significantly affect detected community structures (Table 1), and sieved and unsieved sediment resolved comparable communities at the phylum-level (Fig. 3), although community segregation was observed in ordinations of metazoans resolved with 18S V1–V2 and protists resolved with 18S V4 (Fig. 4). Between 21 and 36% of sediment OTUs/ASVs were shared among sieved and unsieved sediment samples. Shared metazoan OTUs primarily belonged to Hydrozoa (18S, 2.5%, COI, 32%, Siphonophorae, Anthoathecata, Leptothecata), Demospongiae (COI, 9%), Gastropoda (COI, 32%), Nematoda (18S, 61% Chromadorea, 11% Enoplea), Polychaeta (18S, 2.5%), or Copepoda (18S, 4.5%). Microbial ASVs shared among sieved and unsieved sediment mostly belonged to Syndiniales (17%), Filosa (19%), Ciliophora (11%), Dinophyceae (9%), Planctomycetes (22%), Acidobacteria (10%), or Proteobacteria (Gamma, 9%, Alpha, 8%, Delta, 11%).

In contrast, sampling method significantly affected resolved community structure for water, as for all size fractions, the sampling box detected significantly different communities than the in situ pump (Table 1). Both sampling methods resolved different communities at the phylum-level (Fig. 3), and water samples always clustered apart in ordinations (Fig. 4) for all taxonomic compartments investigated.. Between 8 and 11% of ASVs/OTUs detected in water were shared between the in situ pump and the sampling box. Taxonomic structures resolved by both sampling methods clearly changed due to targeted size fraction. The sampling box’s 2–20 µm size fraction did not detect the same metazoan community assemblage as the > 20 µm assemblage detect by the pump (Fig. 3 COI and 18S V1–V2). Similarly, for microbial data, the in situ pump targeting the > 20 µm size class, and the sampling box targeting both the 2–20 µm and the 0.2–2 µm size classes, detected different community assemblages. For protists, the in situ pump detected higher proportions of ASVs for Bacillariophyta, Ciliophora, Labyrinthulea, or Phaeodarea, while the sampling box detected more cryptophytes, haptophytes, MAST, and telonemians (Fig. 3 18S V4). For prokaryotes, the sampling box detected more diversity in the Alphaproteobacteria, Chloroflexi, or Marinimicrobia (Fig. 3 16S V4–V5).

## Discussion

### Importance of substrate nature

Sediment samples, whether sieved or unsieved, led to the detection of higher numbers of metazoan OTUs and prokaryote ASVs than water samples (Fig. 1), indicating that more diversity could be found in the benthos compared to the pelagos at this Mediterranean site for those groups. For unicellular eukaryotes, the difference in diversity between sediment and aboveground water was not significant. However, this may primarily be due to the fact that more benthic protists (e.g., filosans, labyrinthuleans, and ciliates) were well detected by water samples (Supplementary Fig. S2 online). Indeed, 19% of protist sediment ASVs were also detected in the water samples, while for other loci this percentage was at 5–8%. These findings are congruent with other studies in the marine realm that reported notably higher diversity in sediments compared to seawater^13–15^ for microbial communities, and show that higher diversity can also be expected for metazoans.

Community compositions differed markedly between sediment and aboveground water samples for all life compartments investigated (Figs. 3, 4), and only 3–8% of total molecular clusters were shared between substrate types, a range congruent with previous findings^11,15,16^. Metazoan infauna taxa (e.g., nematodes, platyhelminths, kinorhynchs, tardigrades, and xenacoelomorphs) were specifically detected by sediment samples, while other epibenthic, benthopelagic, and pelagic metazoans were more prevalent in water samples (e.g., echinoderms, chordates, ctenophores). Similarly, with protists and prokaryotes, sediment samples detected lineages typically reported in the deep seafloor, with prokaryotic communities mostly comprised of Proteobacteria, Acidobacteria, Planctomycetes, Archeae, Bacteroidetes, and Chloroflexi^17–20^, and protist communities characterized by benthic heterotrophic groups such as ciliates, labyrinthuleans, and filosans^21,22^. Water samples instead recovered taxa commonly reported in pelagic studies, with unicellular eukaryotes such as dinoflagellates (Dinophyceae, Syndiniales), radiolarians (Acantharea, Phaeodarea, Spumellarida), or MAST (incl. diatoms, Chlorophyta, Chrysophyceae)^11,23,24^, and bacterial groups such as Alpha- and Gamma-proteobacteria, Bacteroidetes and Cyanobacteria^25–27^.

Most of the metazoans shared among sediment and water samples displayed benthopelagic life cycles with a benthic adult and a pelagic larva (hydrozoans, gastropods, demosponges, polychaetes), indicating that the detection of benthic taxa in water samples may predominantly reflect the occurrence of dispersal phases of those organisms^10^. Other metazoan OTUs shared across sediment and water belonged to Copepoda (incl. Cyclopoida and Harpacticoida), Polychaeta, and Nematoda, confirming that active dispersal and/or resuspension of benthic taxa can also occur^9^. Similarly, benthic protists (e.g. filosans, labyrinthuleans), and bacteria known to occur at the sediment–water interface (e.g., Bacteroidetes, Planctomycetes)^14,28^, were also predominant in this shared fraction, supporting the existence of sediment resuspension dynamics. Finally, the presence of pelagic taxa such as Actinopteri, scyphozoans, cephalopods, diatoms (Bacillariophyta), and dinoflagellates (Dinophyceae and parasitic Syndiniales) in both sediment and water samples supports the fact that dead material, detritus, or faecal pellets can sink to the deep seafloor^29^.

Overall, our results confirm previous findings showing that sample nature strongly affects the type of organisms targeted by eDNA metabarcoding^30,31^, and underline that eDNA from water samples cannot be used to comprehensively survey benthic communities^16,32,33^, even when large volumes of aboveground water are collected.

### Sieving sediment is not essential for comprehensive benthic biodiversity surveys

Studies investigating the effect of size-sorting in macroinvertebrates showed that sorting organisms by size and pooling them proportionately according to their abundance led to a more equal amplification of taxa, the sorted samples recovering 30% more taxa than the unsorted samples at the same sequencing depth^34^. The size fractions used in this study were specifically aiming to concentrate the meiofauna (32 µm–1 mm) compartment, which is known to be important in deep-sea sediments, both in terms of abundance and biomass^2,35,36^. Meiofauna taxa, best captured by 18S V1–V2, were more numerous in sieved than unsieved sediment samples, and total recovered OTU numbers were higher in sieved than in unsieved samples for two cores with this marker (Fig. 1). These differences were however not significant. It could be that the equimolar pooling performed with DNA extracts from each different size fraction maintained biases in abundance, as larger organisms contributed more DNA molecules within each size fraction. Alternatively, some individual size fractions having yielded low DNA concentrations, their equimolar pool effectively diluted the size fractions that generated most of the DNA. Indeed, highest DNA recovery was observed in the 20–40 µm and the 40–250 µm size fractions for all cores, while the larger size fractions had DNA concentrations < 1 ng/µL. This problem was particularly severe for PL11, explaining why this core performed so poorly in the sieved treatment for both 18S markers (Fig. 1). Proportional pooling may be a better approach, but is feasible only if relative abundance of organisms in each size class can be calculated (e.g., using dry sample and specimen weights). A more accurate approach would be to sequence each size fraction separately, which would likely result in many more ASVs/OTUs due to increased sequencing depth. This however would also increase sequencing costs five-fold. An alternative to sequencing each size fraction separately could be the pooling of the size fractions after PCR, which would reduce size related biomass biases during PCR amplification, without increasing sequencing costs. However, the fact that more diversity was detected when sieving than when not sieving at the same sequencing depth for the 18S marker (Supplementary Fig. S4 online), indicates that sieving effectively reduces biomass biases, thus allowing the detection of more diversity at the same sampling depth. Alternatively, new technologies affording much higher sequencing depths^37^ might circumvent the need for size-class sorting in the future.

The advantage provided by sieving observed in this study for some phyla may also result from the fact that sieved samples were based on more starting material, as five DNA extractions were performed for the sieved treatment (one for each size fraction), when only one was performed for non-sieved sediment. Sample volume can however not fully explain differences in recovered diversity, as the latter varied considerably within each method, and samples based on larger sediment volumes did not consistently yield more OTUs/ASVs (Fig. 1, Supplementary Fig. S3 online).

Elutriation (i.e. resuspension of organisms and pouring of supernatant on a 32-µm sieve) or density extraction techniques are other methods traditionally used to study meiofauna^38,39^. These allow to process whole sediment layers more rapidly than sieving, and effectively concentrate metazoan organisms^38^. However, if the retention of organisms is achieved using only a single mesh size marking the lower size boundary of meiofauna, this also maintains size-abundance biases. Thus, whether sieving, elutriating, or density extracting, mesh sizes for size-class sorting have to be carefully chosen in order to reach the best compromise between processing time and biomass biases. As underlined by Elbrecht et al. (2017)^34^, sorting is most useful when samples contain specimens with biomasses spanning several orders of magnitude. Given that deep-sea sediments contain large numbers of small organisms, and given the high detection capacity of metabarcoding, implementing five mesh sizes for sorting metazoans may be excessive. Instead, separating organisms into small, medium, and large size categories, as performed by Elbrecht et al. (2017)^34^ for freshwater macroinvertebrates and by Leray & Knowlton (2015)^40^ for coastal benthic communities may be sufficient to maximize metazoan species detection.

However, the rationale behind size sorting should be carefully considered when implementing an eDNA metabarcoding study on the deep seafloor. Indeed, for general biodiversity studies not targeting rare of invasive species, the proportion of abundant taxa is most relevant to reach accurate conclusions, and it may thus not be necessary to detect all small and rare taxa in such studies. Moreover, effects of size sorting on other taxonomic compartments have to be taken into consideration. For microbial organisms, sieving down to a 20-µm mesh size is very likely to result in the loss of most small and/or free-living taxa. This idea is supported by the fact that metazoan OTUs shared between sieved and unsieved sediment were mainly assigned to macrofauna (> 1 mm), indicating that small taxa predominantly explain the differences obtained between both methods. For protists and prokaryotes, although sieved and unsieved sediment uncovered comparable alpha diversity levels (Fig. 1), and resolved similar taxonomic compositions at phylum level (Fig. 3), ordinations indicated that communities segregated with processing method for protists (Fig. 4 18S V4). Many sediment microorganisms are living within biofilms (e.g., Bacteroidetes, Archeae), attached to sediment particles (e.g., Planctomycetes) or as symbionts of larger taxa (e.g., Syndiniales, some Dinophyceae and Proteobacteria), making their retention on a 20-µm sieve possible. Our results support this idea, as microbial ASVs shared among sieved and unsieved sediment were mostly belonging to those groups or to taxa larger than 20 µm (e.g. ciliates), possibly explaining the non-significant difference we obtained in PERMANOVA (Table 1).

Finally, sieving is associated to higher contamination risks, as sieves need to be carefully washed between samples and water used for sieving (or elutriation) needs to be ultra-filtered (which can be problematic for the large volumes needed). Considering the limited improvement gained by sieving on metazoan communities, the logistic inconvenience, and the risk of bias for other taxonomic compartments, DNA extractions performed directly on 10 g of sediment appear as a satisfactory approach for large-scale biodiversity surveys targeting multiple life compartments.

### Adjusting water sample volume and filter mesh size to target organisms

Numerous aquatic metabarcoding studies have highlighted that sampled water volume is a key factor affecting species detection rates with eDNA, and has to be adapted to the target ecosystem^41^. Positive relationships between increased water volume and increased detection rate have been reported for macroinvertebrates and amphibians^42,43^, and studies in freshwater ecosystems have shown that 20 l to 30–68 l of water are necessary to detect entire metazoan communities^12,44,45^. While 1 L may be appropriate for macroinvertebrate detection in rivers^42^ or marine surface waters^46^, the results presented here clearly show that 7.5 L of deep-sea water are not sufficient to accurately detect metazoan fauna. The sampling boxes detected less metazoan diversity than the in situ pump (Fig. 1), and failed to detect many phyla with 18S V1-V2 (Fig. 2). Overall, 21% (COI) to 39% (18S V1–V2) of metazoan diversity detected in water was recovered by the sampling box, compared to 89% (COI) and 71% (18S V1–V2) by the in situ pump. This reflects the low abundance and biomass of large organisms in deep waters, combined with the very limited lifetime of extracellular DNA in seawater^47–50^.

Water sampling methods for eDNA metabarcoding relying on on-board filtration or precipitation are intrinsically limited by the amount of water that can be processed. Although purpose-built sampling equipment has been developed for increased efficiency and standardization, filtration flow rates rarely exceed 1 L/min^51^. New developments allowing the processing of thousands of litres of water, such as the SALSA in situ pump presented here, its equivalent single-sample version^52^, or tow net methods developed for lentic ecosystems^53^, improve the detection sensitivity for metazoan taxa in low biomass environments and will allow for more comprehensive and reliable surveys.

With protists and bacteria, taxonomic structures recovered by each sampling method clearly changed with targeted size class (Fig. 3, Supplementary Fig. S2 online). Most protistan micro- to mesoplankton were better detected by the in situ pump (e.g., diatoms, phaeodareans, Acantharea, Ciliophora), while pico- to nanoplankton were preferentially targeted by the sampling box (e.g., Haptophyta, Telonemia), with many groups identified mostly by the smallest size fraction (0.2–2 µm, Chlorophyta, Choanoflagellida, Picozoa, Chrysophyceae, MAST). For bacteria, groups known to occur in aggregates, on larger particles, or as symbionts of larger organisms were better recovered by the in situ pump (e.g., Actinobacteria, Bacteroidetes, Delta-, Gammaproteobacteria, Lentisphaerae, Firmicutes, Tenericutes), while other, likely free-living, bacterioplankton were predominant in the sampling box samples (e.g., Cyanobacteria, Marinimicrobia). This differential taxon recovery of water collection methods has already been reported in shallower studies^24^, and highlights the importance of targeting the 0.2–2 µm for accurately surveying microbial diversity.

Although the SALSA prototype presented here (Supplementary Fig. S1 online) has since been improved to pump through a 5-µm nylon mesh, in situ filtration techniques are inherently limited by mesh size in order to filter large volumes of water. Thus, although targeting large volumes such as the ones allowed by SALSA represents the most suitable strategy for assessing metazoan diversity in deep-sea waters, its limitation in terms of mesh size leads to the detection of only a fraction of microbial diversity, i.e. mostly larger planktonic groups or taxa fixed on larger faunal specimens or mineral particles. On board filtration of smaller volumes of water remains necessary to access the pico- and nanoplankton, highlighting that both sampling methods are complementary and should be deployed in parallel for integrative biodiversity surveys across the tree of life.

Overall, this comparative study contributes to more comprehensive and more reliable assessments of metazoan and microbial deep-sea communities based on eDNA metabarcoding. First, only sediment samples can allow the characterization of benthic taxa and aboveground water samples do not provide a good alternative. Second, sieving sediment leads to an improvement in taxon detection for metazoans, but as expected, also modifies the retrieved community composition for protists and prokaryotes. Thus, for studies targeting only metazoans, it is advisable to first separate the organisms from the sediment particles using sieving, elutriation, or density extraction techniques as recommended by Brannock & Halanych^38^. If both metazoan and microbial communities are targeted, and provided sample volume is large enough, an ideal sampling design would be to use multiple sub-samples for microbial taxa and size-sort the remaining sediment for detecting metazoans, as suggested by Nascimento et al.^54^. Alternatively, as shown here, using sufficient volumes of unsorted sediment seems to be satisfactory for integrative biodiversity studies across taxonomic compartments. Finally, water sample volume and mesh size need to be carefully chosen depending on taxa of interest, and while volumes collected by sampling boxes (or Niskin bottles) allow the surveying of microbial diversity, much larger volumes are needed to detect deep-sea metazoans.

## Methods

### Sample collection

Sediment cores and water samples were collected from a continental slope site during the EssNaut16 cruise in the Mediterranean in April 2016 (Supplementary Table S1 online). Sampling was carried out with a human operated vehicle (Nautile, Ifremer). Triplicate tube cores were collected at the sampling site, and the upper first centimetre sediment layer was used to compare two sediment sampling methods. The sediment samples were either (1) transferred into zip-lock bags and frozen at − 80 °C on board or (2) sieved through five different mesh sizes (1000 µm, 500 µm, 250 µm, 40 µm, and 20 µm) in order to concentrate organisms and separate them by size-class. Sieving was performed with cold surface water filtered at 0.2 µm. Each mesh concentrate was subsequently stored in a separate zip-lock bag and frozen at − 80 °C. All samples were shipped on dry ice to the laboratory.

Two different aboveground water-sampling methods were evaluated during EssNaut16 to target microbial and metazoan taxa. All water samples were collected at most 1 m above the seafloor. Water was collected with a newly developed in situ pump, the Serial Autonomous Larval Sampler (SALSA), i.e. a McLane WTS-LV sampler adapted by Ifremer, Brest, France to allow replicated sampling. SALSA has a rosette holding five 2.8 L sampling bowls mounted underneath a rotator plate that allows the alignment of each sampling bowl with the water intake, in a pre-programmed sequential fashion (Supplementary Fig. S1 online). The pump is placed downstream the sampling bowls and the outlet of each bowl is equipped with a 20-µm nylon mesh, retaining particles larger than 20 µm within the bowl while the water passes through the outlet. SALSA thus allows to obtain a time-series of five samples, each resulting from a 4 h filtration event that concentrates particles from ~ 6000 L of water (depending on the filtration rate applied) at the exact same location. Here, 2 samples of each time series were used while the remaining samples were sorted for classical morphological diversity assessments. Two SALSA deployments were performed at the study site (PL07, PL11) and one deployment within the same habitat but at shallower depth due to technical reasons impeding deployment at the original site (PL09). Analyses were performed with and without PL09, and as results were comparable, PL09 was included in the study. For each SALSA deployment, particles retained in every sample (i.e. 2.8 L sampling bowl containing > 20 µm particles retained during in situ filtration) were concentrated on board on a polycarbonate filter membrane with 2-µm mesh size (Millipore, Burlington, MA, USA, ref. TTTP04700). Water was also collected using two 7.5 L Nautile-deployed sterile and watertight sampling boxes^30^. These samples were filtered on board successively through membrane filters with 20 µm, 2 µm, and 0.2 µm mesh size (Millipore, Burlington, MA, USA, refs. NY2004700, TTTP04700, GTTP04700), generating three size fractions (> 20 µm, 2–20 µm, and 0.2–2 µm). Each water filter was stored in an individual Petri dish, frozen at − 80 °C, and shipped on dry ice to the laboratory.

### Nucleic acid extractions

For sediment, DNA extractions were performed using 2–10 g of sediment (Supplementary Table S1 online) with the PowerMax Soil DNA Isolation Kit (MOBIO Laboratories Inc.; Qiagen, Hilden, Germany). All DNA extracts were stored at − 80 °C. For the non-sieved method, DNA was extracted directly from 2 to 5 g of sediment, the volume varying with the amount of sediment available. For the sieved method, DNA was extracted from each size fraction separately (from 1 to 10 g of sediment per size fraction), and for each of the three cores, an equimolar pool of the DNA extracts of each size fraction was prepared for PCR and sequencing. Thus, for the sieved method, the samples, were based on 3–6 times more sediment than the unsieved raw extracts, however, as some size fractions yielded low DNA concentrations in each core, the sieved samples were at a lower DNA concentration than the unsieved samples (Supplementary Table S1 online). Water DNA extractions were carried out by Genoscope (Évry, France) using the same protocol as described by Alberti et al. (2017)^55^ for Tara Oceans water samples. The protocol is based on cryogenic grinding of membrane filters, followed by nucleic acid extraction with NucleoSpin RNA kits combined with the NucleoSpin DNA buffer set (Macherey–Nagel, Düren, Germany). A negative extraction control was performed alongside sample extractions for both water and sediment samples, adding nothing in the place of sample in the first extraction step.

### PCR amplification and sequencing

DNA extracts were normalised to 0.25 ng/µL and 10 µL of standardized sample were used for PCR. Four primer pairs were used to amplify one mitochondrial and three ribosomal RNA (rRNA) barcode loci. The cytochrome c oxidase I (COI)^56,57^ and 18S V1-V2 rRNA^8^ barcodes were used to target metazoans, while 18S V4^58^ was used for unicellular eukaryotes, and 16S V4–V5^59^ for prokaryotes (Supplementary Table S2 online). PCR amplifications for each locus (see supplemental information for amplification and purification details) were carried out in triplicate in order to level-off intra-sample variance while obtaining sufficient amounts of amplicons for Illumina sequencing. PCR triplicates were pooled, purified, quality checked and quantified, and 100 ng of amplicons were directly end-repaired, A-tailed, and ligated to Illumina adapters on a Biomek FX Laboratory Automation Workstation (Beckman Coulter, Brea, CA, USA). Library amplification was performed using a Kapa Hifi HotStart NGS library Amplification kit (Kapa Biosystems, Wilmington, MA, USA), with the same cycling conditions applied for all metagenomic libraries, and libraries were purified using 1X AMPure XP beads (Beckman Coulter, Brea, CA, USA). They were then quantified by Quant-iT dsDNA HS assay kits using a Fluoroskan Ascent microplate fluorometer (Thermo Fisher Scientific, Waltham, MA, USA) and sample amplicon libraries were pooled equimolarly. The pools were then quantified by qPCR with the KAPA Library Quantification Kit for Illumina Libraries (Kapa Biosystems, Wilmington, MA, USA) on an MxPro instrument (Agilent Technologies, Santa Clara, CA, USA). Library profiles were assessed using a high-throughput microfluidic capillary electrophoresis system (LabChip GX, Perkin Elmer, Waltham, MA, USA). , Libraries normalised to 8–9 pM and containing a 20% PhiX spike-in were sequenced individually on HiSeq2500 (System User Guide Part # 15035786) instruments in a 250 bp paired-end mode. For sediment, this procedure was carried out on two DNA aliquots of each sample (for each core, 2 aliquots of not sieved extract and 2 aliquots of “sieved pool”), leading to two amplicon libraries per sample. For water collected with the sampling box, we targeted and sequenced microbial loci in the 0.2–2 and 2–20 µm fractions, and targeted metazoans in the 2–20 and > 20 µm fractions. The size fractions were processed separately but sequencing failed for the > 20 µm fractions with microbial loci, possibly due to the low DNA concentrations of these samples.

### Bioinformatic analyses

All bioinformatic analyses were performed using a Unix shell script, available on Gitlab (https://gitlab.ifremer.fr/abyss-project/), on a home-based cluster (DATARMOR, Ifremer), and the samples of the present study were analysed in parallel with 12 to 28 other deep-sea water samples for more accurate error correction and LULU filtering. The details of the pipeline, along with specific parameters used for all metabarcoding markers, are given in Supplementary Table S3 online.

Illumina read pairs were corrected with DADA2 v.1.10^60^, following the online tutorial for paired-end data (https://benjjneb.github.io/dada2/tutorial.html), delivering inventories of Amplicon Sequence Variants (ASVs). We chose to evaluate unicellular eukaryote and prokaryote diversity at the ASV level due to their lower intraspecific diversity, making ASVs appropriate to study species-level biodiversity patterns in these microbial taxa. Intraspecific diversity being much more pronounced in metazoans than unicellular organisms due to the extremely varying numbers of cells, organelles, pseudogenes (e.g. numts for COI), or ribosomal repeats in their genomes, ASVs reflect metazoan diversity at the intra-species level, which is dependent on the level of intraspecific variation in the genome, known to vary widely among taxa^61,62^. As we were interested in species-level diversity, we chose to cluster metazoan data. ASVs from COI and 18S V1-V2 were clustered into Operational Taxonomic Units (OTUs) with swarm v2^63^ using the FROGS pipeline^64^. Swarm v2 is a single-linkage clustering algorithm that aggregates sequences iteratively and locally around seed sequences based on *d*, the number of nucleotide differences, to determine coherent groups of sequences. This avoids a universal clustering threshold, which is particularly useful in highly biodiverse samples such as those analysed in this study. Metazoan ASVs were swarm-clustered at *d* = *3* for 18S V1–V2 (~ 99%) and *d* = *6* for COI (~ 98%), which has been shown to be appropriate for evaluating species diversity in samples^65^.

Clusters were taxonomically assigned with BLAST + (v2.6.0) based on minimum similarity (70%) and minimum coverage (80%). For ASVs, sequences obtained with DADA2 were subsequently assigned with *blastn.* For OTUs, BLAST assignment was performed in FROGS using the *affiliation_OTU.py* command. The Silva132 reference database was used for taxonomic assignment of the 16S V4–V5 and 18S V1–V2 rRNA marker genes^66^, PR2 v4.11^67^ was used for 18S V4, and MIDORI-UNIQUE^68^ reduced to marine taxa only was used for COI. An initial test implementing BLAST + to assign taxonomy only to the COI dataset using a 96% percent identity threshold led to the exclusion of the majority of the clusters. Indeed, it is not uncommon for deep-sea taxa to have closest relatives in databases (even congenerics) exhibiting nucleotide divergences of 20%^69,70^.Considering our interest in diverse and poorly characterized communities, more stringent BLAST thresholds were not implemented at this stage. However, additional filters were performed during downstream bioinformatic processing described below.

Molecular inventories were refined in R v.3.5.1^71^. A blank correction was made using the *decontam* package v.1.2.1^72^, removing all clusters that were more prevalent in negative control samples (PCR and extraction controls) than in true samples. After comparison, results from the technical duplicates produced for sediment samples were merged and read counts were summed for identical OTUs. Fully unassigned clusters were removed (COI: 63%, 18S V1–V2: 20%, 18S V4: 17%, 16S: 5%). When present, non-target clusters were removed (protists or fungi in 18S V1–V2: 60%; metazoans or plants in 18S V4: 10.5%). Additionally, for COI and 18S V1–V2, all metazoan OTUs with a terrestrial assignment (groups known to be terrestrial-only) were removed (COI: 1.5%, 18S: 0.15%). Samples were checked to ensure they had more than 10,000 target reads. Metazoan OTU tables were further curated with LULU v.0.1^73^ to limit bias due to intraspecific variation and pseudogenes, using a minimum co-occurrence of 0.95, a minimum match at 84%, and a minimum ratio at 1000, which is more appropriate for sample-poor datasets^62^.

### Statistical analyses

Data were analysed using R with the packages phyloseq v1.22.3^74^, following guidelines in online tutorials (http://joey711.github.io/phyloseq/tutorials-index.html), and vegan v2.5.2^75^. Read and cluster abundances were evaluated via analyses of variance (ANOVA) on generalised linear models using quasipoisson distributions. Pairwise post-hoc comparisons were performed via Tukey HSD tests using the *emmeans* package. Alpha and beta diversity were compared among sampling methods using datasets rarefied to the minimum sequencing depth (COI: 62,660; 18S V1: 127,044; 18S V4: 37,000; 16S: 100,952). For comparisons by phylum, paired Welch’s t-tests were performed for comparing both sediment methods, and unpaired t-tests were performed for other comparisons. If normality was not verified (Shapiro–Wilk normality test), Wilcoxon (paired) rank tests were performed. Differences in community composition among methods were assessed with Venn diagrams (computed using the *venn* function in the venn package) and with permutational multivariate analysis of variance (PERMANOVA), using the *adonis2* function (vegan) with significance evaluated using 1000 permutations. Incidence-based Jaccard dissimilarities were used for metazoans, while Bray–Curtis dissimilarities were used for prokaryotes and unicellular eukaryotes. The rationale behind this choice is that metazoans are multicellular organisms of extremely varying numbers of cells, organelles, or ribosomal repeats in their genomes, and can also be detected through a diversity of remains. The number of reads can thus not be expected to reliably reflect the abundance of detected OTUs. Pairwise PERMANOVAs among sampling methods were performed with the *pairwiseAdonis* package. Differences in community structures among samples were visualized via detrended correspondence analyses on rarefied incidence datasets. Finally, taxonomic compositions in terms of cluster abundance were compared among processing methods only using clusters reliably assigned at phylum-level. Phylum-level reliability thresholds were chosen based on Stefanni et al.^76^ and were set at minimum hit identity of 86% for rRNA loci and 80% for COI.

## Supplementary Information

Supplementary Information 1.

Supplementary Information 2.

Supplementary Information 3.

## Data Availability

The raw data for this work can be accessed in the European Nucleotide Archive database (Study accession numbers: PRJEB37673 for water, PRJEB33873 for sediment). Please refer to the metadata excel file for ENA file names. The dataset, including raw sequences, databases, as well as raw and refined ASV/OTU tables are available on https://doi.org/10.12770/2deb785a-74c5-4b9d-84d6-82a81e0dda6d. Bioinformatic scripts can be accessed following the Gitlab link.
