# Supplementary material for: Evaluating sediment and water sampling methods for the estimation of deep-sea biodiversity using environmental DNA
Source: Sci Rep. 2021 Apr 12;11:7856. doi: 10.1038/s41598-021-86396-8 (PMC8041860; doi:10.1038/s41598-021-86396-8)
Supplement: Supplementary file 1 — Supplementary Information 1. [file 41598_2021_86396_MOESM1_ESM.pdf]

Brandt MI, Pradillon F, Trouche B, Henry N, Liautard-Haag C, Cambon-Bonavita MA, Cueff-Gauchard V, Belser C,Wincker P, Poulain J, Arnaud-Haond S, and Zeppilli D. 2021. Evaluating sediment and water sampling methods for the estimation of deep-sea biodiversity using environmental DNA.

SUPPLEMENTARY MATERIAL

Supplemental materials and methods

Eukaryotic 18S V1-V2 rRNA gene amplicon generation

Eukaryotic 18S V1-V2 barcodes were generated using the SSUF04 (5'-GCTTGTCTCAAAGATTAAGCC-3') and SSUR22*mod* (5'-CCTGCTGCCTTCCTTRGA-3') primers and the *Phusion* High Fidelity PCR Master Mix with GC buffer (ThermoFisher Scientific, Waltham, MA, USA). The PCR reactions (25 µL final volume) contained 2.5 ng or less of DNA template with 0.4 µM concentration of each primer, 3% of DMSO, and 1X Phusion Master Mix.

PCR amplifications (98 °C for 30 s; 25 cycles of 10 s at 98 °C, 30 s at 45 °C, 30 s at 72 °C; and 72 °C for 10 min) of all samples were carried out in triplicate in order to smooth the intra-sample variance while obtaining sufficient amounts of amplicons for Illumina sequencing. Amplicon triplicates were pooled and PCR products were purified using 1X AMPure XP beads (Beckman Coulter, Brea, CA, USA) clean-up. Aliquots of purified amplicons were run on an Agilent Bioanalyzer using the DNA High Sensitivity LabChip kit (Agilent Technologies, Santa Clara, CA, USA) to check their lengths, and quantified with a Qubit fluorometer (Invitrogen, Carlsbad, CA, USA).

Eukaryotic 18S V4 rRNA gene amplicon generation

Eukaryotic 18S V4 barcodes were generated using the TAReukF1 (5'-CCAGCASCYGCGGTAATTCC-3') and TAReukR (5'-ACTTTCGTTCTTGATYRA-3')

primers. Triplicate PCR reactions were prepared as described above, but amplification was performed by a nested PCR with the first annealing temperature being 53°C for 10 cycles, followed by 48°C for 15 cycles. After PCR product clean-up using 1X AMPure XP beads, amplicon lengths and amounts were checked as described above.

Prokaryotic 16S V4-V5 rRNA gene amplicon generation

Prokaryotic barcodes were generated using the 515F-Y (5′-GTGYCAGCMGCCGCGGTAA-3′) and 926R (5′- CCGYCAATTYMTTTRAGTTT-3′**)** primers. Triplicate PCR reactions were prepared as described above for 18S V1-V2, but annealing temperature was at 53 °C. After PCR product clean-up using 1X AMPure XP beads, amplicon lengths and amounts were checked as described above.

Eukaryotic COI gene amplicon generation

Metazoan COI barcodes were generated using the mlCOIintF 5'-GGWACWGGWTGAACWGTWTAYCCYCC-3'and jgHCO2198 5'-TAIACYTCIGGRTGICCRAARAAYCA-3' primers. The PCR reactions (20 µL final volume) contained 2.5 ng or less of total DNA template with 0.5 µM final concentration of each primer, 3% of DMSO, 0.175 mM final concentration of dNTPs, and 1X Advantage 2 Polymerase Mix (Takara Bio, Kusatsu, Japan). Nested PCR amplifications were carried out in triplicate and consisted of an initial denaturation at 95 °C for 10 min, and 16 cycles of 10 s at 95°C, 30 s at 62 °C (−1°C per cycle), 60 s at 68 °C followed by 15 cycles of 95 °C for 10 s, 30 s at 46°C, 68 °C for 60 s, and a final extension of 68 °C for 7 min.

Supplemental tables

Table S1. Sampling sites, their GPS locations, and associated habitats. Sieved sediment was sieved through five mesh sizes (1,000; 500; 250; 40; 20 µm), and DNA was extracted from each size fraction separately. An equimolar pool of the five DNA extracts of each size fraction was then made for PCR and sequencing of the sieved samples. Volume for PCR was always 10 µl, for template stock standardized at ≤ 0.25 ng/µl.

Sample name	ENA sample alias	Extraction kit	Sampling method	Size fraction (µm)	Sample volume for DNA extraction	Concentration of original extract (ng/µL)	Depth (m)	Latitude	Longitude	Habitat	Region
ESSNAUT_PL06_CT2_0_1_rep1	eDNAB0000081	PowerMax Soil DNA Isolation Kit	Not sieved	NA	2 g	1.1	2,417	42.9422	6.7422	Continental slope	Gulf of Lyon, Western Mediterranean
ESSNAUT_PL06_CT2_0_1_rep2	eDNAB0000081	PowerMax Soil DNA Isolation Kit	Not sieved	NA	2 g	1	2,417	42.9422	6.7422	Continental slope	Gulf of Lyon, Western Mediterranean
ESSNAUT_PL07_CT2_0_1_rep1	eDNAB0000122	PowerMax Soil DNA Isolation Kit	Not sieved	NA	5.1 g	3.2	2,415	42.9423	6.7426	Continental slope	Gulf of Lyon, Western Mediterranean
ESSNAUT_PL07_CT2_0_1_rep2	eDNAB0000122	PowerMax Soil DNA Isolation Kit	Not sieved	NA	5.1 g	3	2,415	42.9423	6.7426	Continental slope	Gulf of Lyon, Western Mediterranean
ESSNAUT_PL11_CT2_0_1_rep1	eDNAB0000189	PowerMax Soil DNA Isolation Kit	Not sieved	NA	4 g	2.7	2,418	42.9423	6.7423	Continental slope	Gulf of Lyon, Western Mediterranean
ESSNAUT_PL11_CT2_0_1_rep2	eDNAB0000189	PowerMax Soil DNA Isolation Kit	Not sieved	NA	4 g	2.6	2,418	42.9423	6.7423	Continental slope	Gulf of Lyon, Western Mediterranean
ESSNAUT_PL06_CT4_0_1_pool_rep1	eDNAB0003158	PowerMax Soil DNA Isolation Kit	Sieved	>1,000; 500-1,000; 250-500; 40-250; 20-40	2.2+0.7+1.5+2+2.2=7.6 g	0.4	2,417	42.9422	6.7422	Continental slope	Gulf of Lyon, Western Mediterranean
ESSNAUT_PL06_CT4_0_1_pool_rep2	eDNAB0003158	PowerMax Soil DNA Isolation Kit	Sieved	>1,000; 500-1,000; 250-500; 40-250; 20-40	2.2+0.7+1.5+2+2.2=7.6 g	0.6	2,417	42.9422	6.7422	Continental slope	Gulf of Lyon, Western Mediterranean
ESSNAUT_PL07_CT4_0_1_pool_rep1	eDNAB0003159	PowerMax Soil DNA Isolation Kit	Sieved	>1,000; 500-1,000; 250-500; 40-250; 20-40	1.4+1.2+1.5+10+10=24.1 g	0.9	2,415	42.9423	6.7426	Continental slope	Gulf of Lyon, Western Mediterranean
ESSNAUT_PL07_CT4_0_1_pool_rep2	eDNAB0003159	PowerMax Soil DNA Isolation Kit	Sieved	>1,000; 500-1,000; 250-500; 40-250; 20-40	1.4+1.2+1.5+10+10=24.1 g	1.2	2,415	42.9423	6.7426	Continental slope	Gulf of Lyon, Western Mediterranean
ESSNAUT_PL11_CT4_0_1_pool_rep1	eDNAB0003160	PowerMax Soil DNA Isolation Kit	Sieved	>1,000; 500-1,000; 250-500; 40-250; 20-40	0.7+0.4+5.4+6.9+10=23.4 g	0.3	2,418	42.9423	6.7423	Continental slope	Gulf of Lyon, Western Mediterranean
ESSNAUT_PL11_CT4_0_1_pool_rep2	eDNAB0003160	PowerMax Soil DNA Isolation Kit	Sieved	>1,000; 500-1,000; 250-500; 40-250; 20-40	0.7+0.4+5.4+6.9+10=23.4 g	0.3	2,418	42.9423	6.7423	Continental slope	Gulf of Lyon, Western Mediterranean
EssNaut_DNA_extraction blank	eDNAB0003157	PowerMax Soil DNA Isolation Kit	Blank	NA	NA	0.2	NA	NA	NA	NA	NA
Water_extraction_blank	eDNAB0003316	Tara Oceans extraction protocol	Blank	NA	NA	0.2	NA	NA	NA	NA	NA
ESSNAUT_PL11_Salsa3Bol2_20	eDNAB0000222	Tara Oceans extraction protocol	in situ pump	> 20	6,300 L	3.02	2,417	42.9425	6.7440	Continental slope	Gulf of Lyon, Western Mediterranean
ESSNAUT_PL11_Salsa3Bol4_20	eDNAB0000224	Tara Oceans extraction protocol	in situ pump	> 20	6,300 L	3.22	2,417	42.9425	6.7440	Continental slope	Gulf of Lyon, Western Mediterranean
ESSNAUT_PL7_Salsa1Bol3_20	eDNAB0000149	Tara Oceans extraction protocol	in situ pump	> 20	4,740 L	6.96	2,417	42.9423	6.7426	Continental slope	Gulf of Lyon, Western Mediterranean
ESSNAUT_PL7_Salsa1Bol5_20	eDNAB0000150	Tara Oceans extraction protocol	in situ pump	> 20	4,740 L	5	2,417	42.9423	6.7426	Continental slope	Gulf of Lyon, Western Mediterranean
ESSNAUT_PL9_Salsa2Bol2_20	eDNAB0000155	Tara Oceans extraction protocol	in situ pump	> 20	5,400 L	16.3	1,152	43.2237	6.8876	Continental slope	Gulf of Lyon, Western Mediterranean
ESSNAUT_PL9_Salsa2Bol4_20	eDNAB0000157	Tara Oceans extraction protocol	in situ pump	> 20	5,400 L	6.46	1,152	43.2237	6.8876	Continental slope	Gulf of Lyon, Western Mediterranean
ESSNAUT_PL10_PBT2_0.2	eDNAB0002506	Tara Oceans extraction protocol	Sampling box	0.2-2.0	7.5 L	3.6	2,420	42.9425	6.7444	Continental slope	Gulf of Lyon, Western Mediterranean
ESSNAUT_PL10_PBT2_2	eDNAB0002507	Tara Oceans extraction protocol	Sampling box	2.0-20	7.5 L	0.01	2,420	42.9425	6.7444	Continental slope	Gulf of Lyon, Western Mediterranean
ESSNAUT_PL10_PBT2_20	eDNAB0002508	Tara Oceans extraction protocol	Sampling box	> 20	7.5 L	0.01	2,420	42.9425	6.7444	Continental slope	Gulf of Lyon, Western Mediterranean
ESSNAUT_PL7_PBT2_0.2	eDNAB0002503	Tara Oceans extraction protocol	Sampling box	0.2-2.0	7.5 L	1	2,417	42.9423	6.7426	Continental slope	Gulf of Lyon, Western Mediterranean
ESSNAUT_PL7_PBT2_2	eDNAB0002504	Tara Oceans extraction protocol	Sampling box	2.0-20	7.5 L	0.01	2,417	42.9423	6.7426	Continental slope	Gulf of Lyon, Western Mediterranean
ESSNAUT_PL7_PBT2_20	eDNAB0002505	Tara Oceans extraction protocol	Sampling box	> 20	7.5 L	0.01	2,417	42.9423	6.7426	Continental slope	Gulf of Lyon, Western Mediterranean

Table S2. Primers used in this study, targeting metazoans with the COI and 18S V1-V2 loci, unicellular eukaryotes with the 18S V4 locus, and prokaryotes with the 16S V4-V5 marker.

**Locus**	**Target and *specificity***	**Primer forward Primer reverse**	**Short name**	**Sequence (5'-3')**	**Amplicon size (bp)**	**Reference**
**COI**	Eukaryotes	mlCOIintF	COI-F	GGWACWGGWTGAACWGTWTAYCCYCC	313	Leray et al., 2013
	pref. metazoans	jgHCO2198	COI-R	TAIACYTCIGGRTGICCRAARAAYCA		
**18S V1-V2**	Eukaryotes	SSUF04	18S-V1F	GCTTGTCTCAAAGATTAAGCC	330-390	Sinniger et al., 2016
	pref. metazoans	SSURmod	18S-V1-R	CCTGCTGCCTTCCTTRGA		
**18S V4**	Eukaryotes	V4F (TAReukFWD1)	18S-V4-F	CCAGCASCYGCGGTAATTCC	350-410	Stoeck et al., 2010
	all	V4R (TAReukREV3)	18S-V4-R	ACTTTCGTTCTTGATYRA		
**16S V4-V5**	Prokaryotes	515f	16S-F	GTGYCAGCMGCCGCGGTAA	350-390	Parada et al., 2016
	Pref. Eubacteria	926r	16S-R	CCGYCAATTYMTTTRAGTTT		

Table S3. ABYSS metabarcoding pipeline

Process	Software	Script(s) and command(s)
Raw reads preprocessing for ligation data	Abyss-preprocessing: separate forward and reverse reads in each run, and re-pair reads	extract.sh using extractR1R2.py with cutadapt v1.18 (-e 0.14-0.17 for 18S,16S i.e. 3 nt mismatches and 0.27 for COI i.e. 7 nt mismatches , -O length of primer -1) and BBMAP Repair v38.22
Read quality-filtering	Dada2 v.1.10	filterAndTrim() in dada2main.R maxEE=2, maxN=0, truncQ=11, truncLen=220 (18S, 16S) or 200 (COI)
Read error learning	Dada2 v.1.10	learnErrors() in dada2main.R nbases=1e8, multithread=TRUE, randomize=TRUE
Read dereplicating	Dada2 v.1.10	derepFastq() in dada2main.R
Read correction	Dada2 v.1.10	dada() in dada2main.R
Read merging	Dada2 v.1.10	mergePairs() in dada2main.R minOverlap=12, maxMismatch=0
Make sequence table and filter by length	Dada2 v.1.10	makeSequenceTable() in dada2main.R seqtab[,nchar(colnames(seqtab)) %in% seq(lengthMin,lengthMax)] lengthMin= 330 (18S-V1), 300 (COI), 350 (18S-V4), 350 (16S) lengthMax= 390 (18S-V1), 326 (COI), 410 (18S-V4), 390 (16S)
Chimera removal	Dada2 v.1.10	removeBimeraDenovo() in dada2main.R
Taxonomic assignment with RDP Classifier	Dada2 v.1.10	assignTaxonomy() in dada2outputfiles.R minBoot=50, outputBootstraps=TRUE
Taxonomic assignment of ASVs with BLAST+	blastn (megablast) v.2.6.0	blast.pbs -outfmt 11 -qcov_hsp_perc 80 -perc_identity 70 -max_hsps 1, -evalue 1e-5, then merge BLAST and RDP taxonomies using concat_blast_rdp_tax.pbs
Clustering of ASVs, chimera removal, taxonomic assignment (optional)	FROGS v.2.0.0	frogs.pbs using clustering.py with d=3 for 18S V1-V2 and d=6 for COI, then remove_chimera.py, and affiliation_OTU_identite_couverture.py
Blank correction	Rscript	Data_refining.Rmd using packages decontam v.1.2.1 and phyloseq v.1.26.0
Removal of unassigned and non-target clusters		
Deletion of defective samples (< 10,000 target reads)		
LULU curation	LULU v.0.1	lulu.R using minimum_ratio_type = "min", minimum_ratio = 1000, minimum_match = 84 minimum_relative_cooccurence = 0.95

Table S4. Number of reads and clusters (ASVs for 18S V4 and 16S, OTUs for 18S V1-V2 and COI) obtained at different analysis steps, depending on sample processing category. Data refining was performed in R, based on BLAST assignments obtained using the Silva v132 database for 18S V1-V2 and 16S loci, on the PR2 database for 18S V4, and on the MIDORI marine-only database for COI. Final number of target reads represent the number of target-taxa reads after data refining (decontamination, removal of unassigned and non-target clusters). Final number of target clusters are the corresponding ASVs for 18S V4 and 16S, and the corresponding OTUs for 18S V1-V2 and COI, after additional LULU curation for those two loci.

Sample type	Number of samples	Raw reads	Quality-filtered reads	Merged reads	Length-filtered reads	Non chimeric reads	% reads retained	Number of raw clusters	Total raw clusters	Final number of target reads	Final number of target clusters
**LOCUS**											
**COI**											
Sampling box	4	5,633,675	4,972,882	4,924,011	4,882,680	4,837,720	86	1,103	10,351	8,475,278	2,319
in situ pump	6	5,904,107	5,560,956	5,457,180	5,038,842	4,770,288	81	2,709			
Not sieved sediment	6	5,139,091	4,395,309	4,327,685	3,877,338	3,855,547	75	4,609			
Sieved sediment	6	5,210,192	4,384,663	4,332,251	3,702,371	3,671,540	70	3,844			
PCR Control Sample	6	465,237	262,309	255,630	254,827	254,817	55	78		na	na
Extraction Control Sample	2	3,233,904	2,475,388	2,431,972	2,426,469	2,417,278	75	29		na	na
**18S V1-V2**											
Sampling box	2	2,079,109	1,850,870	1,817,310	1,759,578	1,735,231	83	906	17,608	5,744,248	1,460
in situ pump	6	5,357,877	4,709,302	4,367,841	4,238,624	3,981,561	74	4,659			
Not sieved sediment	6	3,890,833	2,665,878	2,463,769	2,050,489	2,034,698	52	4,699			
Sieved sediment	5	4,719,584	3,621,503	3,329,633	3,077,489	3,047,637	65	4,984			
PCR Control Sample	4	1,976,483	1,123,546	1,080,187	914,275	913,588	46	199		na	na
Extraction Control Sample	2	1,434,680	1,143,161	1,082,658	729,709	712,577	50	153		na	na
**18S V4**											
Sampling box	4	4,142,505	3,691,442	3,647,381	3,645,131	3,597,953	87	5,176	35,538	4,793,443	8,031
in situ pump	6	6,055,792	5,355,958	5,204,132	5,203,747	4,883,139	81	6,905			
Not sieved sediment	6	1,190,150	855,544	742,230	731,661	728,993	61	5,572			
Sieved sediment	5	1,655,300	1,219,956	1,030,018	1,020,309	1,016,622	61	5,380			
PCR Control Sample	4	5,153	1,014	867	867	867	17	21		na	na
Extraction Control Sample	2	1,097,221	848,500	838,436	837,909	825,498	75	174		na	na
**16S V4-V5**											
Sampling box	4	3,434,755	2,952,132	2,623,265	2,606,614	2,517,523	73	7,363	62,646	7,024,386	39,961
in situ pump	6	3,987,382	3,148,002	1,219,715	1,169,737	1,154,763	29	16,567			
Not sieved sediment	6	3,544,156	2,431,818	2,095,989	2,083,590	1,995,875	56	29,553			
Sieved sediment	6	2,829,692	1,952,664	1,612,822	1,608,064	1,540,155	54	29,128			
PCR Control Sample	4	1,516,645	1,327,867	1,324,078	1,320,742	1,320,640	87	125		na	na
Extraction Control Sample	2	2,095,233	1,677,554	1,428,751	1,398,492	1,397,637	67	268		na	na

Supplemental figures

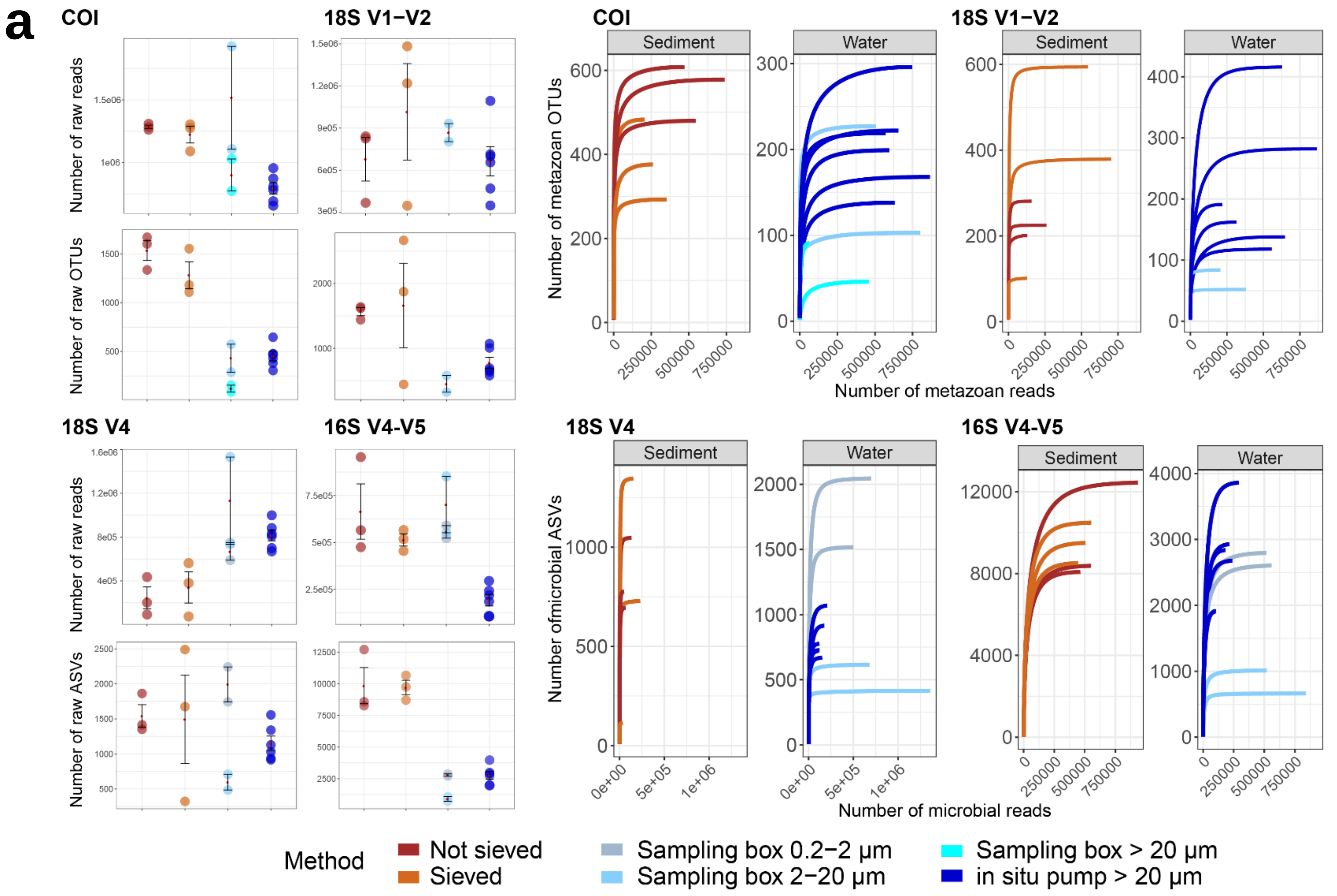

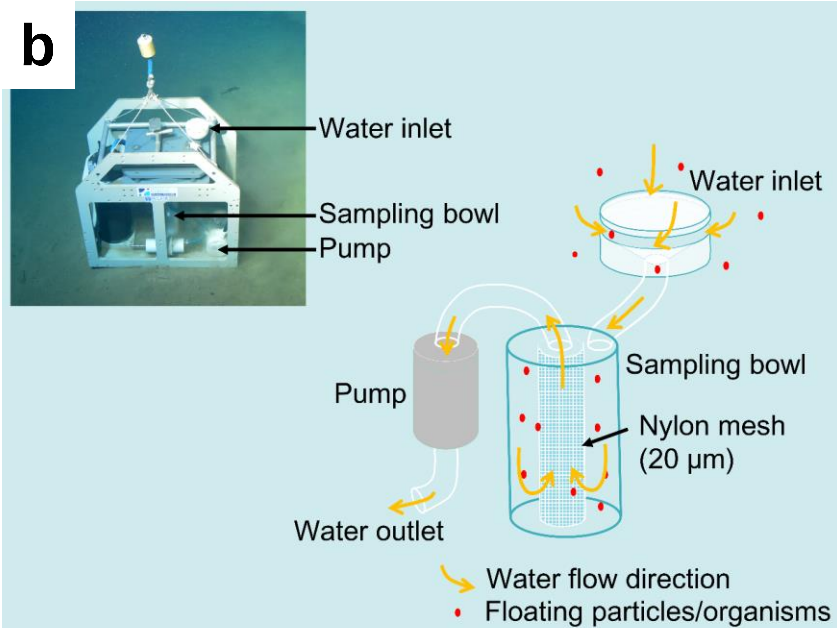

Figure S1. **(a)** Raw read and cluster numbers in deep-sea sediment (brown) and aboveground water (blue) with different sampling methods in metabarcoding inventories of metazoans (COI, 18S V1-V2), micro-eukaryotes (18S V4), and prokaryotes (16S V4-V5). Sediment was either sieved through 5 mesh sizes to size-sort organisms prior DNA extraction, or DNA was extracted directly from crude sediment samples. Water was collected with a 7.5 L sampling box, allowing recovery of up to two size classes in each taxonomic compartment, or sampled in large volumes with an in situ pump. Rarefaction curves were performed on refined datasets and show a plateau is reached in most samples, except sediment samples with 18S V4, and in situ pump samples for both microbial loci. **(b)** SALSA in situ pump prototype diagram.

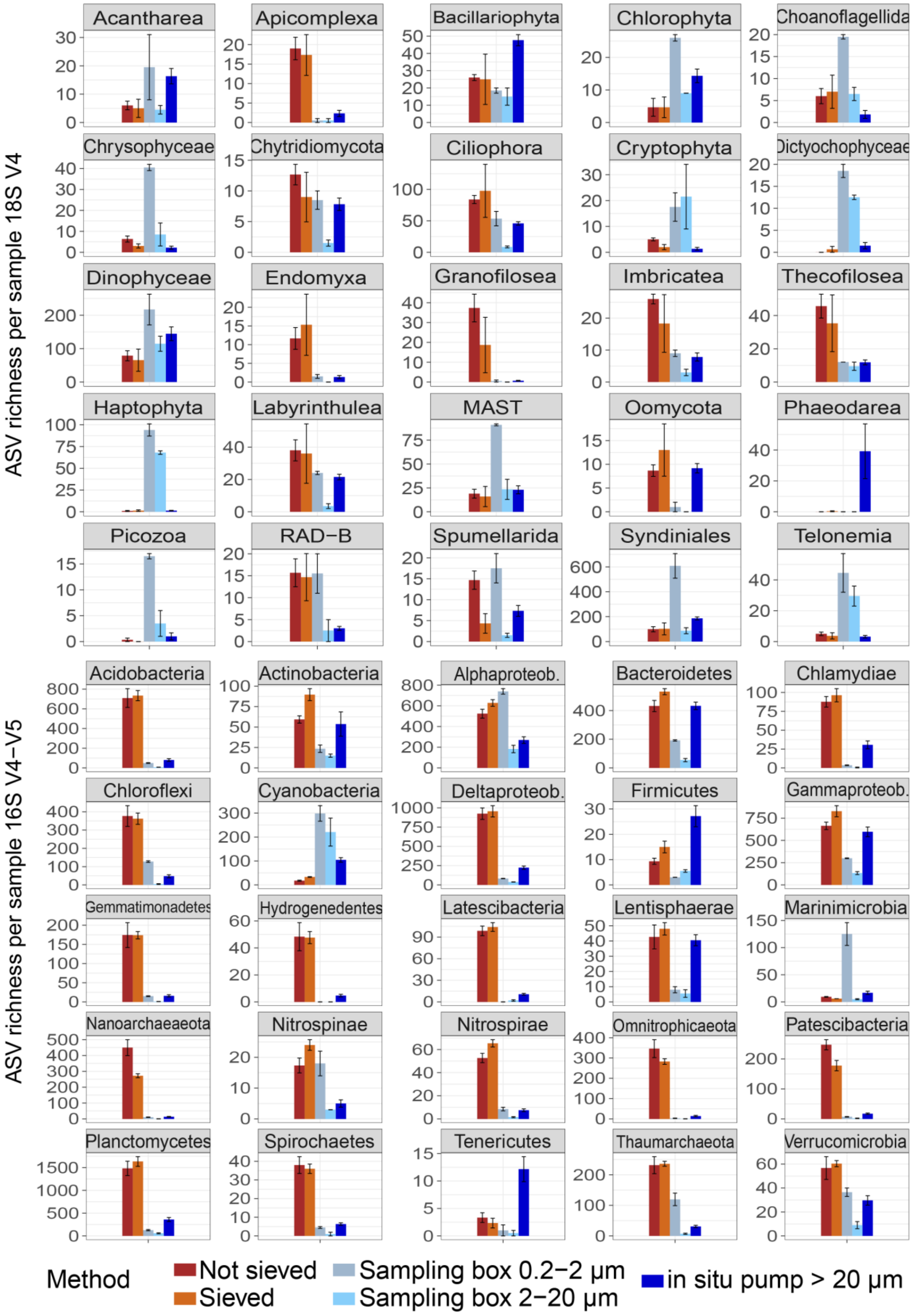

Figure S2. Mean numbers (±SE) of protist (18S V4) and prokaryote (16S V4-V5) Amplicon Sequence Variants (ASVs) in major taxonomic lineages, recovered by deep-sea sediment (brown) and aboveground water (blue), using two sampling methods for both sample types. Sediment was either sieved to size-sort organisms prior DNA extraction, or DNA was extracted directly from crude sediment samples. Water was collected with a 7.5 L sampling box, allowing recovery of two size classes, or sampled in large volumes with an *in situ* pump. ASV numbers were calculated on the rarefied datasets.

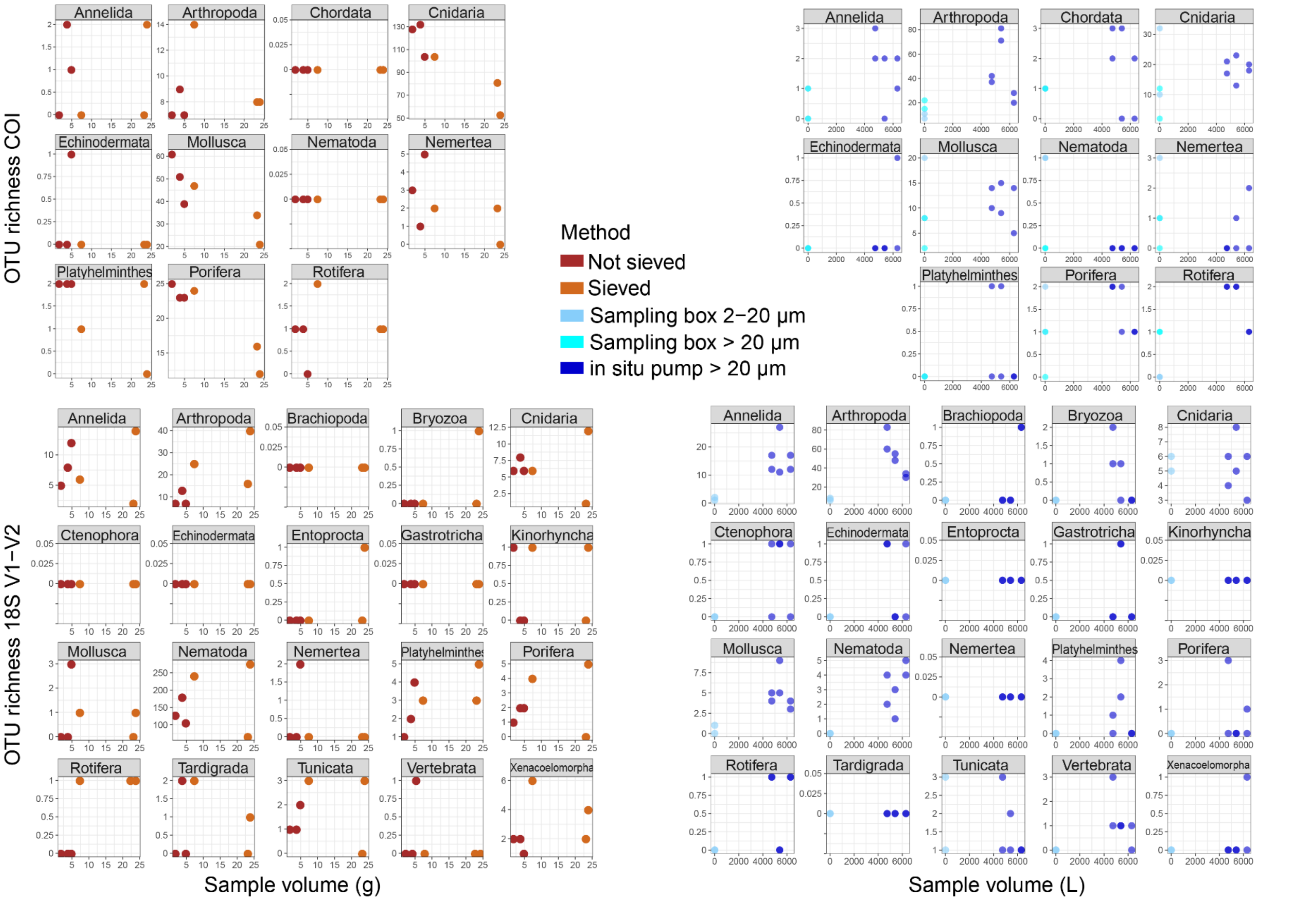

Figure S3. Numbers of metazoan OTUs detected with COI (top) and 18S V1-V2 (bottom) in deep-sea sediment (brown) and aboveground water (blue), using for each sample type two sampling methods based on varying amounts of starting material. Sediment was either sieved through 5 mesh sizes to size-sort organisms prior DNA extraction, or DNA was extracted directly from crude sediment samples. Water was collected with a 7.5 L sampling box, allowing recovery of up to two size classes per taxonomic compartment, or sampled in large volumes with an *in situ* pump. Cluster abundances were calculated on rarefied datasets.

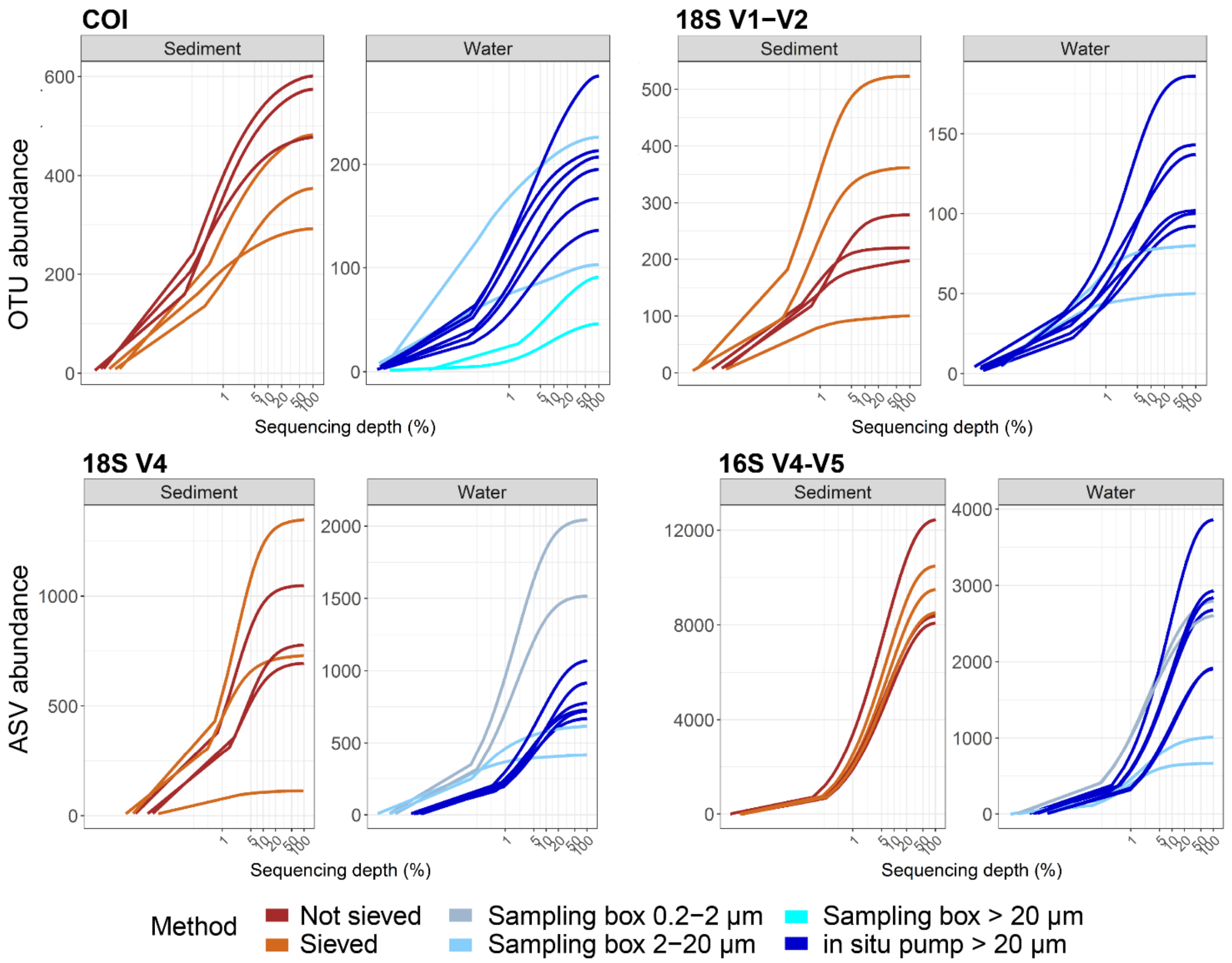

Figure S4. Number of metazoan OTUs (COI, 18S V1-V2) and microbial ASVs (18S V4, 16S V4-V5) recovered at different levels of sequencing depths in deep-sea sediment (brown) and aboveground water (blue), using two sampling methods for both sample types. Sediment was either sieved to size-sort organisms prior DNA extraction, or DNA was extracted directly from crude sediment samples. Water was collected with a 7.5 L sampling box, allowing recovery of up to two size classes per taxonomic compartment, or sampled in large volumes with an *in situ* pump. The sequencing depth is plotted on a logarithmic scale.
